# A 14-Marker Multiplexed Imaging Panel for Prognostic Biomarkers and Tumor Heterogeneity in Head and Neck Squamous Cell Carcinoma

**DOI:** 10.3389/fonc.2021.713561

**Published:** 2021-08-19

**Authors:** Junichi Mitsuda, Takahiro Tsujikawa, Kanako Yoshimura, Sumiyo Saburi, Masaho Suetsugu, Kayo Kitamoto, Mari Takenaka, Gaku Ohmura, Akihito Arai, Hiroshi Ogi, Kyoko Itoh, Shigeru Hirano

**Affiliations:** ^1^Department of Otolaryngology–Head and Neck Surgery, Kyoto Prefectural University of Medicine, Kyoto, Japan; ^2^Department of Cell, Developmental & Cancer Biology, Oregon Health and Science University, Portland, OR, United States; ^3^Department of Pathology and Applied Neurobiology, Kyoto Prefectural University of Medicine, Kyoto, Japan; ^4^SCREEN Holdings Co., Ltd., Kyoto, Japan

**Keywords:** meta-analysis, biomarker, immunohistochemistry, tumor heterogeneity, head and neck squamous cell carcinoma (HNSCC)

## Abstract

Recent advances made in treatment for head and neck squamous cell carcinoma (HNSCC) highlight the need for new prediction tools to guide therapeutic strategies. In this study, we aimed to develop a HNSCC-targeting multiplex immunohistochemical (IHC) panel that can evaluate prognostic factors and the intratumor heterogeneity of HNSCC. To identify IHC-based tissue biomarkers that constitute new multiplex IHC panel, a systematic review and meta-analysis were performed to analyze reported IHC biomarkers in laryngeal and pharyngeal SCC in the period of 2008–2018. The Cancer Genome Atlas (TCGA) and Reactome pathway databases were used to validate the prognostic and functional significance of the identified biomarkers. A 14-marker chromogenic multiplex IHC panel including identified biomarkers was used to analyze untreated HNSCC tissue. Forty-five high-quality studies and thirty-one candidate tissue biomarkers were identified (N = 7062). Prognostic validation in TCGA laryngeal and pharyngeal SCC cohort (N = 205) showed that β-catenin, DKK1, PINCH1, ADAM10, and TIMP1 were significantly associated with poor prognosis, which were related to functional categories such as immune system, cellular response, cell cycle, and developmental systems. Selected biomarkers were assembled to build a 14-marker panel, evaluating heterogeneity and polarized expression of tumor biomarkers in the tissue structures, which was particularly related to activation of Wnt/β-catenin pathway. Integrated IHC analysis based on a systemic review and meta-analysis provides an *in situ* proteomics tool to assess the aggressiveness and intratumor heterogeneity of HNSCC.

## Introduction

The emergence of molecular targeting therapy and immune checkpoint blockade has revolutionized the clinical management of head and neck squamous cell carcinoma (HNSCC), but its mortality is still high at approximately 50%, leading to 330,000 deaths per year worldwide (1). Increased number of available therapeutic options urgently requires optimization of the sequence and combination of treatment modalities, thus requiring new tools for the prediction of therapeutic outcomes.

Immunohistochemistry (IHC) has served as the gold standard for histopathological evaluation of cancer. Since a large number of IHC-based biomarkers have been reported, integrative organization of reported biomarkers is needed to identify clinically meaningful biomarkers related to disease aggressiveness of HNSCC. However, systematic reviews and meta-analyses of IHC-based biomarkers have not been carried out for HNSCC, particularly for pharyngeal and laryngeal SCC, where decision making for treatment of these disease is directly associated with organ preservation, including voice and swallowing function.

Intratumor heterogeneity has been increasingly acknowledged since technological advances in single-cell sequencing methods were made, where the presence of subpopulations of cancer cells with distinct genotypic, phenotypic, and morphological profiles was observed within a single tumor, including HNSCC (2–4). Since intratumor heterogeneity is strongly associated with therapeutic response and resistance (5), better treatment strategies can be developed based on the characterization of intratumor heterogeneity. Given that transcriptomic profiles do not highly correlate with proteomic changes (6), intratumor heterogeneity needs to be evaluated at both the transcription and protein levels, highlighting the significance of revisiting IHC-based assessment in whole tumor tissue.

Recently, we established a 14-biomarker multiplex IHC platform to enable comprehensive profiling of tumor and immune infiltrates in HNSCC (7). In this study, we performed a systematic review and meta-analysis based on the preferred reporting items for systematic reviews and meta-analyses statement (PRISMA) (8) to identify IHC-based tissue biomarkers associated with the prognosis of pharyngeal and laryngeal SCC. Modified reporting recommendations for tumor marker prognostic studies (REMARK) criteria (9) were utilized for the selection of high-quality studies, followed by prognostic and functional validation using the Cancer Genome Atlas (TCGA) and the Reactome pathway database. This led to the identification of selective biomarkers including β-catenin, DKK1, PINCH1, ADAM10, TIMP1, HIF1α, and ZFX. Based on the above results, we developed a HNSCC-targeting multiplex IHC panel, enabling simultaneous assessment of 14 different tumor and stromal markers with preserved intratumor heterogeneity in a single formalin-fixed paraffin-embedded (FFPE) tissue. In combination with other single-cell and bulk tumor analyses, our chromogenic 14-plex tumor panel can provide spatial information, contributing to the further development of tissue-based biomarkers.

## Materials and Methods

### Systematic Review and Meta-Analysis (PRISMA)

A literature search was performed in the PubMed database (RRID : SCR_004846) on July 7, 2018, using the following search criteria: (head and neck) AND (cancer OR carcinoma) AND (prognos* OR surviv*) AND (immunohistoch*) AND (laryn* OR pharyn*). Two authors independently inspected the titles and abstracts of the retrieved articles between July 2008 and July 2018. Literature written in languages other than English and not analyzed using IHC data were excluded. To provide adequate measures of predictability and improve the quality of the tumor prognostic biomarker studies, modified REMARK criteria containing key items selected by REMARK criteria (9) were used (**Supplementary Table 1**).

The following information was obtained: author name, date of publication, number of patients, IHC protocols, associated multivariate hazard ratio (HR), 95% confidence intervals (CIs) and corresponding P values, overall survival (OS), and evaluated biomarkers. For proteins evaluated in a single study only, the summarized HR (including 95% CI) represented the value reported in that study. For proteins that were evaluated in multiple studies, meta-analysis was carried out using software “R version 3.3.3” and package “metafor”. HR and 95% CI were calculated using both the fixed-effect model and random-effect model. Heterogeneity was evaluated in multiple studies by calculating the Q statistic and I^2^ value. An HR greater than 1 indicated poor prognosis in the study group relative to the reference group and was considered statistically significant if the 95% CI did not overlap. The publication bias was assessed when there were ten or more studies through the inspection of funnel plots.

### *In Silico* Analysis

To evaluate the prognostic significance of the extracted markers, TCGA data (RRID : SCR_003193) was used. The data on 205 laryngeal and pharyngeal SCC cases containing information on the primary site, survival information, OS, and values of biomarker expression were downloaded from the UCSC Xena project (http://xena.ucsc.edu) on September 5, 2019.　Based on the median biomarker expression, all cases were divided into high-expression (N = 102) and low-expression groups (N = 103). The 5-year OS was analyzed using the Kaplan-Meier and log-rank tests. No statistical power analyses were used to determine the sample size. The sample size was based on the number of available and qualified tumor samples for this study. A p value of less than 0.05 was considered statistically significant. The functional significance of the extracted biomarkers was evaluated using the Reactome pathway database (https://reactome.org) (RRID : SCR_003485), where molecular reactions are systematically described in molecular detail to generate a network of molecular transformations (10).

### Multiplex IHC

Sections (5 µm) were obtained from FFPE samples of untreated tongue, oropharyngeal, hypopharyngeal and laryngeal SCC (N = 4). Multiplex IHC was performed as described previously (7). Briefly, FFPE tumor sections were subjected to sequential detection using 14-different antibodies for tumor and stromal markers. Stained slides were digitally scanned using Nanozoomer S60 (Hamamatsu Photonics) at 20 × magnification. Tissue sections were then stripped of antibody and chromogen by cleaning with alcohol and heat-mediated antigen retrieval, followed by subsequent rounds of antibody staining and imaging. A complete list of antibodies and conditions used for staining are provided in **Supplementary Table 2**.

Digital image processing was performed as previously described (7). Digital images reflecting the antibody panel were co-registered and aligned. A sequential gating strategy of image cytometry was applied to identify tumor cell phenotypes based on negative cell staining by using FCS Express 6 Image Cytometry Version 6.03.0011 (De Novo Software). All studies involving human tissues were approved by the Institutional Review Board at Kyoto Prefectural University of Medicine (ERB-C-43-4), and written informed consent was obtained from all patients.

## Results

### A Systematic Review and Meta-Analysis of IHC-Based Studies Identifies 31 Biomarkers Related to Poor Prognosis in Laryngeal and Pharyngeal Cancer

**Figure 1:**
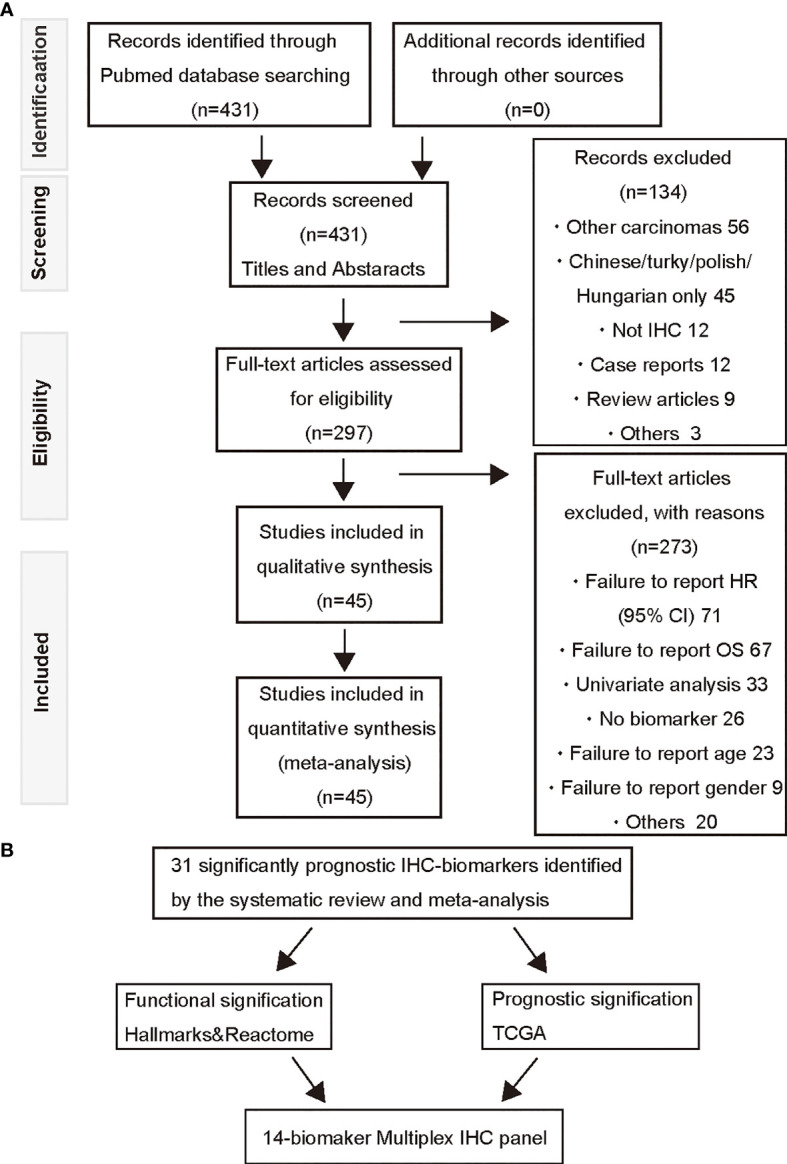
A PRISMA flow diagram of the systematic review of IHC-based prognostic biomarkers and multiplex IHC panel selection. **(A)** A prisma flow diagram presents the PubMed searches, the number of manuscripts excluded, and the identified high-quality cohort studies. **(B)** Selection strategies based on prognostic and functional significance are applied for establishment of the 14-biomaker multiplex IHC panel.

**Figure 2:**
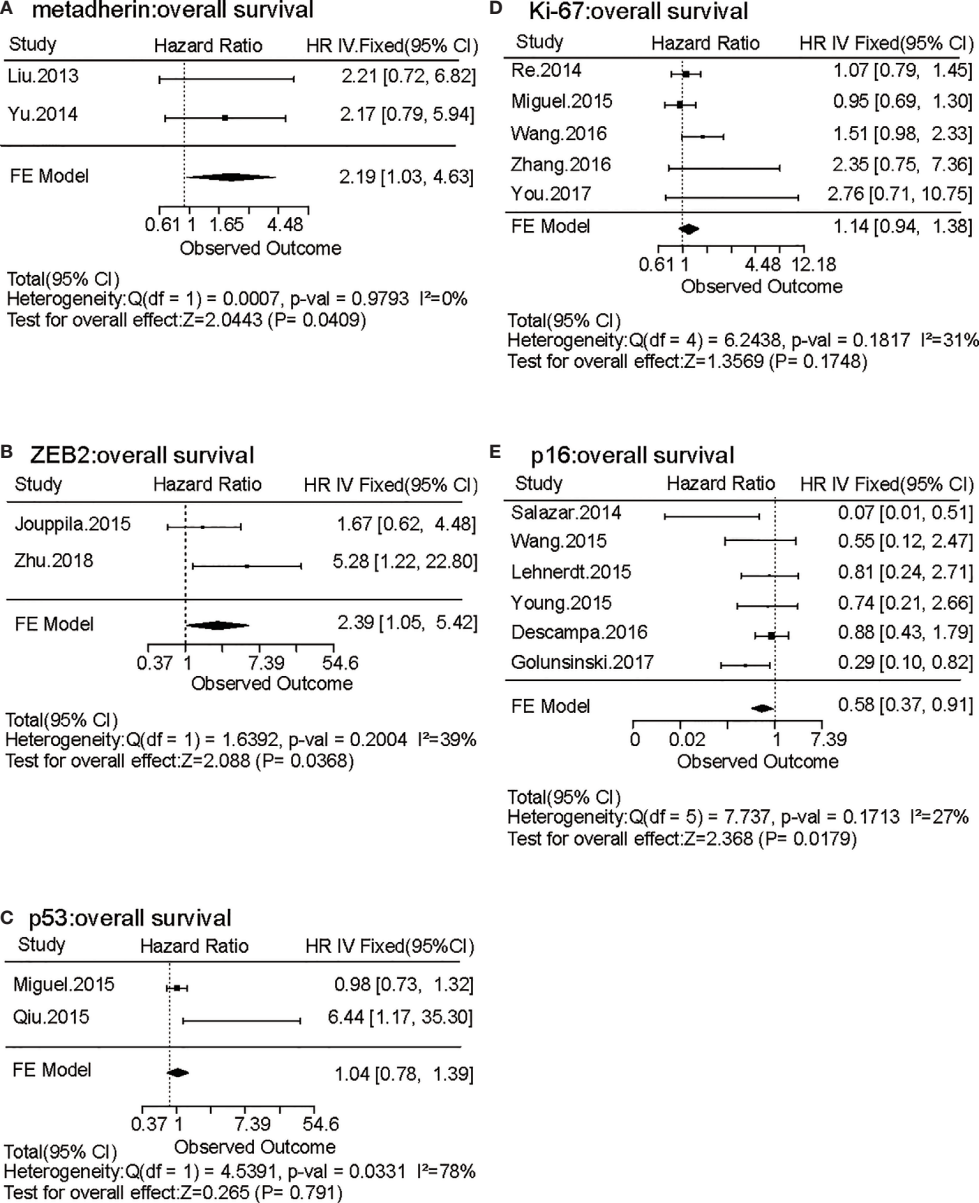
Forest plots of the effects of five biomarkers evaluated by two or more independent studies on laryngeal and pharyngeal HNSCC survival. **(A–E)** Forest plots of hazard ratio (HR) for overall survival (OS) of metadherin **(A)**, ZEB2 **(B)**, p53 **(C)**, Ki-67 **(D)**, and p16 **(E)** are shown based on a fixed effect model. Bars present 95% confidence intervals (CI) of HR, and the center of the lozenge gives the combined HR. Combined fixed effect HRs and tests for heterogeneity (I^2^) are based on the generic inverse variance (I-V) method.

To identify tumor biomarkers that were functionally associated with the prognosis of HNSCC, we performed a systematic review and meta-analysis of previously reported IHC markers in laryngeal and pharyngeal cancers. Our search of the laryngeal or pharyngeal SCC IHC prognostic literature yielded 431 manuscripts for consideration, as shown in **Figure 1**. 56 studies were not related to pharyngeal or laryngeal SCC. 45 studies were written in other languages. 12 manuscripts were excluded, as protein expression was not assessed using IHC. 12 case reports, 9 reviews, 2 meta-analyses, and a preliminary study were excluded. The remaining 297 studies, which used IHC to measure protein expression in laryngeal and pharyngeal SCC, were assessed in terms of study design. Modified REMARK criteria were applied to assess the completeness of reporting (**Supplementary Table 1**). Of the 297 cohort studies, 71 failed to publish the HRs and 95% CIs. 67 studies did not report OS. 33 studies did not perform a multivariable log rank analysis. 26 studies were unrelated to the biomarkers. 32 studies were excluded due to a lack of information such as age, gender. Finally, 45 high-quality cohort studies reporting multivariable-adjusted estimates for 45 biomarkers in laryngeal or pharyngeal SCC were extracted (**Table 1**.** N** = 7062). Since 5 out of 45 biomarkers (metadherin, ZEB2, p53, Ki-67, and p16) were reported by two or more studies, a meta-analysis was performed based on the fixed-effect model (**Figures 2A–E**) and random effect model (**Supplementary Figures 1A–E**), which identified ZEB2 and metadherin as poor prognostic indicators (**Figures 2A, B** **)**. p16 was associated with favorable OS (HR = 0.58, P = 0.017) (**Figure 2E**), in agreement with current clinical management, where p16 was utilized as a highly sensitive surrogate marker for prognostically favorable human papilloma virus-related oropharyngeal cancer (16, 17). Publication bias was not evaluable due to the limited number of studies available for funnel plot inspections (56). Following the exclusion of 5 biomarkers with favorable prognosis and 13 biomarkers that did not meet statistical significance, our systematic review and meta-analysis identified 31 biomarkers that were significantly associated with short OS in laryngeal and pharyngeal HNSCC.

**Table 1:** IHC biomarkers related to overall survival of laryngeal or pharyngeal SCC.

Function	Biomarker	Total No.	HR (95% CI)	P value	Reference
Angiogenesis	EGF-like D7	116	1.74 (1.05-2.17)	0.012	(11)
	HER1	61	1.34 (0.05-72.5)	0.86	(12)
	HIF1α	91	2.56 (1.07-6.15)	0.036	(13)
	TIP30	105	0.331 (0.153-0.715)	0.005	(14)
	VEGF-C	43	0.709 (0.221-2.274)	0.564	(15)
Evading growth suppressor	p16^*^	1495	0.58 (0.37-0.91)	0.017	(16–21)
	p53^*^	182	1.04 (0.78-1.39)	0.791	(22, 23)
	p63	108	1.05 (1.03-1.08)	<0.001	(24)
Genome instability & mutation	MAGE-A9	123	3.57 (1.457-8.762)	0.005	(25)
Immune evasion	β-catenin	76	3.004 (1.24-7.25)	0.014	(26)
Invasion & metastasis	DKK1	102	3.391 (1.46-7.88)	0.005	(27)
	Kif2a	137	2.252 (1.178-4.304)	0.014	(28)
	LAMP1	137	2.17 (1.026-5.996)	0.044	(29)
	LAMP3	117	9.481 (2.216-40.566)	0.002	(22)
	TIMP1	109	2.66 (1.295-5.463)	0.008	(30)
	TROP2	109	2.44 (1.19-5.01)	0.015	(31)
	ZEB2^*^	184	2.39 (1.05-5.42)	0.027	(26, 32)
	Metadherin^*^	365	2.19 (1.03-4.63)	0.04	(33, 34)
Metabolism	GpX1	140	2.101 (1.011-4.367)	0.047	(35)
	GRP78	59	0.875 (0.552-1.335)	0.541	(36)
	pS6	93	0.385 (0.205-0.725)	0.003	(37)
	SGLT	58	1.007 (0.986-1.029)	0.497	(23)
Replicable immortality	FADD	92	0.515 (0.177-1.493)	0.053	(38)
	Geminin	61	4.44 (0.1-224.7)	0.4	(12)
	MAP17	58	0.046 (0.004-0.482)	0.01	(23)
	MCM7	61	6.17 (0.9-67.8)	0.04	(12)
	NDRG3	109	2.863 (1.386-5.915)	0.004	(39)
Resisting cell death	ABCB1	98	1.263 (0.7-2.279)	0.439	(40)
	ABCG2	98	1.744 (1.071-2.839)	0.025	(40)
	KLK6	162	0.358 (0.192-1)	0.001	(41)
	PINCH1	72	2.268 (1.088-4.727)	0.029	(42)
	SIAH	120	0.179 (0.054-0.594)	0.04	(43)
	SOX2	161	1.911 (1.037-3.522)	0.038	(44)
	ZFX	97	3.298 (1.368-7.949)	0.008	(45)
Sustaining proliferation	ASCL1	98	3.165 (1.684-5.952)	<0.05	(46)
	Ki-67^*^	586	1.19 (0.92-1.55)	0.176	(23, 24, 28, 47, 48)
	PHF8	95	2.031 (1.081-3.816)	0.028	(49)
	PTOV1	196	2.806 (1.518-5.185)	0.001	(50)
	SHIP2	54	5.468 (1.459-20.485)	0.012	(51)
	ZNF703	210	1.635 (1.073-2.493)	0.022	(52)
	γ-H2AX	70	6.331 (1.51-28.089)	0.012	(53)
	TNFSF13	198	1.313 (1.027-1.679)	0.003	(48)
Tumor promoting inflammation	ADAM10	78	5.618 (1.188-27.027)	0.03	(47)
	COX-2	80	11.164 (4.628-26.931)	<0.05	(54)
	NF-kB	198	1.254 (1.156-1.36)	<0.05	(55)

*Biomarkers reported by two or more independent studies are marked, where combined summary HRs are calculated by a fixed random effect model (**Figure 2**).

### Functional and Prognostic Significance of Identified IHC Biomarkers Reveals a Broad Spectrum of Tumor Characteristics of HNSCC

The biomarkers identified by the systematic review and meta-analysis were functionally classified into the hallmarks of cancer, such as angiogenesis, evasion of growth suppressors, genome instability and mutation, immune evasion, invasion and metastasis, metabolism, replicable immortality, resisting cell death, sustaining proliferation, and tumor promoting inflammation (57) (**Table 1**). Details of representative markers classified by hallmarks are described in the **Supplementary Information**, showing that prognostic IHC biomarkers were associated with a wide range of hallmarks, particularly with increased invasion and metastasis as well as sustained proliferation. Notably, multiple markers, such as DKK1, ADAM10, and ZFX were related to the Wnt/β-catenin pathway (58–60).

**Figure 3:**
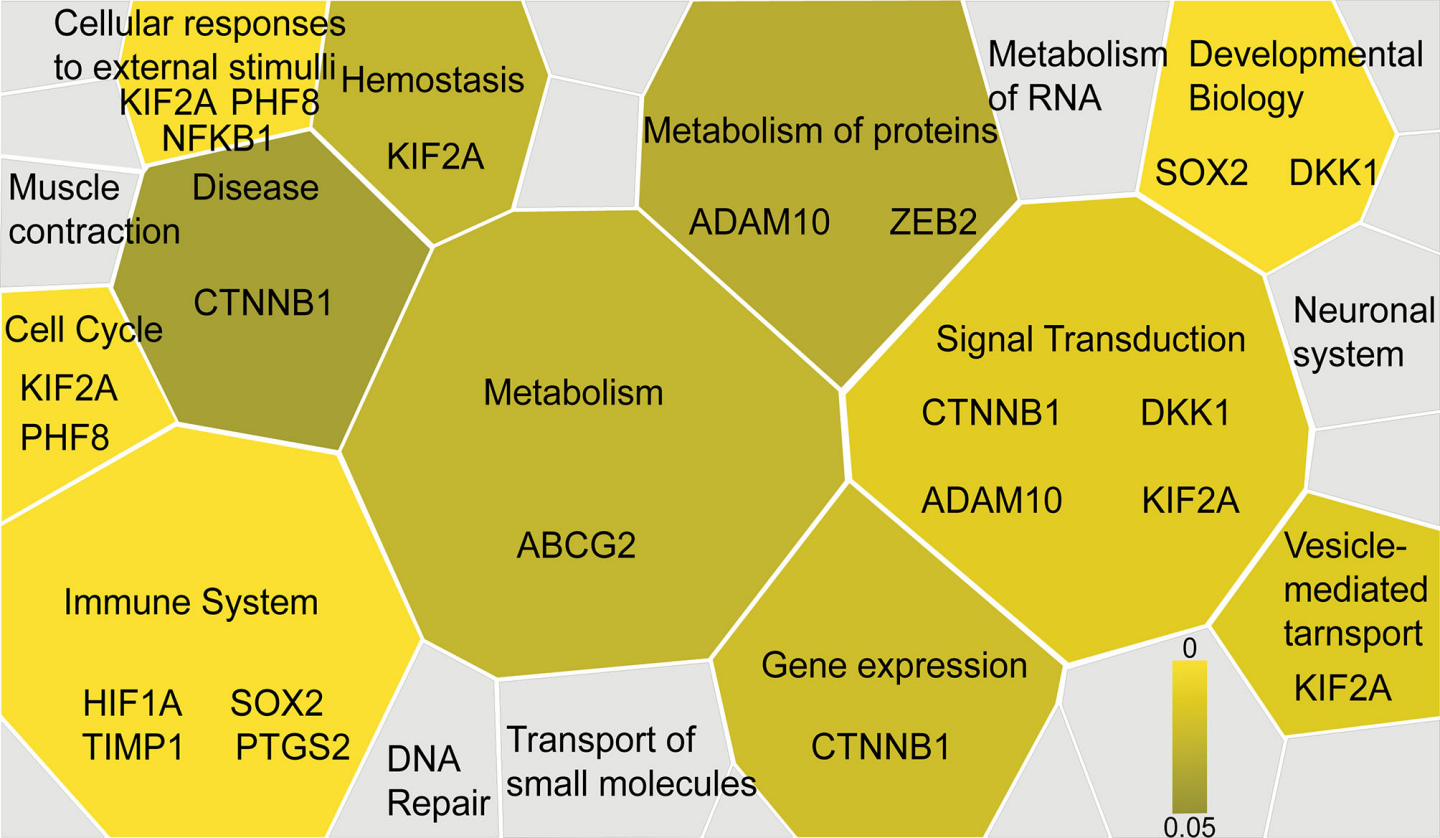
Functional significance of the selected IHC biomarkers identified by the systematic review and meta-analysis. Functional significance of the identified IHC biomarkers is visualized by using the Reactome pathway database (https://reactome.org), reporting on biological pathways associated with dataset of genes. The results are visualized as a Voronoi diagram, showing an overview of the biological pathways related to the 31 biomarkers identified by the systematic review and meta-analysis. Significantly enriched pathways are shown with a color scale from dark to light yellow. IHC biomarkers associated with each pathway are shown.

Furthermore, the functional significance of the identified IHC biomarkers was evaluated using the Reactome pathway database (61), visualizing signaling and metabolic pathways associated with reference gene datasets. A total of 31 genes corresponding to the 31 IHC biomarkers identified by the systematic review and meta-analysis were utilized as a reference dataset, and the results were visualized as a Voronoi diagram based on the ReacFoam function (10), which exhibited an overview of pathways highly related to the identified biomarkers (**Figure 3** and **Supplementary Figure 2** **)**. The immune system, cell cycle, cellular response to external stimuli, and developmental systems were related to the selected 31 biomarkers. β-catenin and related markers were observed in a wide range of functional categories, such as signal transduction, gene expression, and disease, suggesting the possible involvement of Wnt/β-catenin pathways in the progression of HNSCC (**Figure 3** and **Supplementary Figure 2** **)**.

**Figure 4:**
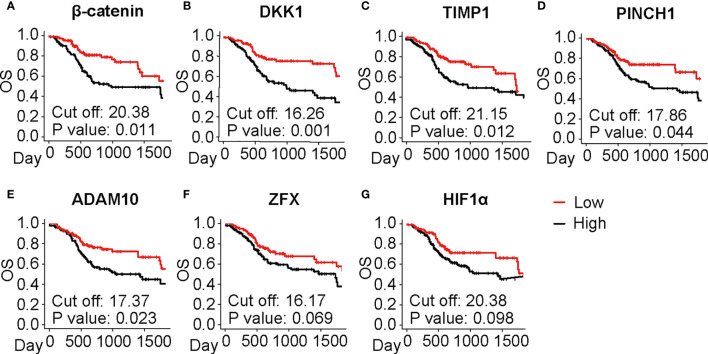
Prognostic significance of the selected IHC biomarkers identified by the systematic review and meta-analysis. **(A–G)** Kaplan-Meier analyses of overall survival (OS) in the laryngeal and pharyngeal HNSCC cohort of the Cancer Genome Atlas (TCGA) (N = 205) stratified by gene expression of the identified biomarkers are shown. Median is used for the cutoff values. Statistical significance is determined using log-rank test.

Since the selected IHC-based biomarkers were related to a broad range of functional properties, we next validated the prognostic significance of these biomarkers using a TCGA cohort in laryngeal and pharyngeal cancer (N = 205). OS was evaluated in relation to representative 31 biomarkers (**Figures 4A–G**, **Supplementary Figures 3A–X** and **4**), showing that β-catenin, DKK1, TIMP1, PINCH1, and ADAM10 were significantly associated with poor prognosis in patients with laryngeal and pharyngeal SCC (**Figures 4A–E**). HIF1α and ZFX tended to be associated with a poor prognosis. Interestingly, high expression of MCM7 or COX2 was associated with favorable prognosis in transcriptome analysis, potentially due to the discrepancy between transcriptional and protein levels. Overall, five biomarkers showed prognostic significance in IHC-based analysis as well as during validation by TCGA analysis, which served as candidates for robust predictive biomarkers for the prognosis of laryngeal and pharyngeal cancer.

### 14-Biomarker Multiplex IHC Panel Highlights Intratumor Heterogeneity in HNSCC

**Figure 5:**
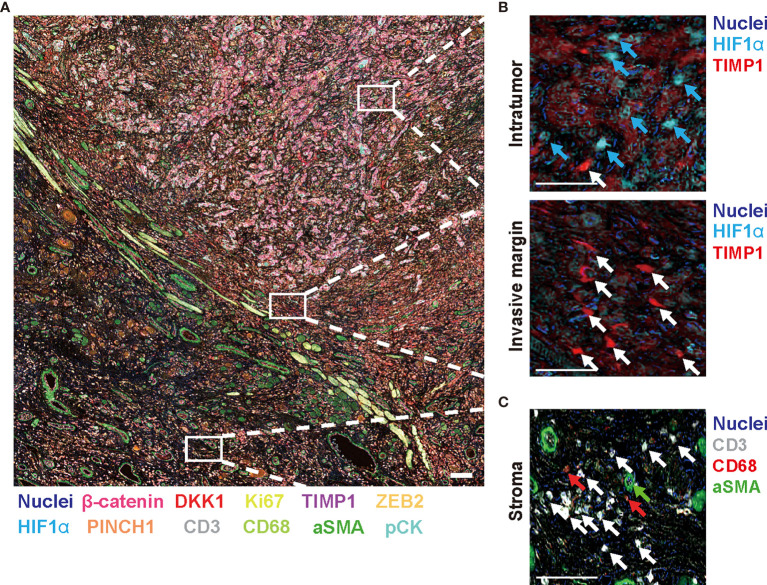
Fourteen-marker multiplex IHC panel to visualize prognostic tumor cell biomarkers and immune/stromal cells in a single FFPE tissue. A FFPE tissue of tongue SCC is visualized by the established 14-marker multiplex IHC. **(A)** Representative 12-color composite images are shown. Biomarkers and colors are shown at the bottom. Scale bar = 100 µm. **(B)** Top and bottom panels present expression of HIF1α and TIMP1 in the intratumor and invasive margin regions, respectively. Blue and white arrows depict HIF1α^+^ cells and TIMP1^+^ cells, respectively. **(C)** Immune markers (CD3 and CD68) and aSMA^+^ cells in stroma region are shown. White, red, and green arrows present CD3^+^, CD68^+^, and aSMA^+^ cells, respectively. Biomarkers and colors are shown at the right. Scale bars = 10 µm. Corresponding single-marker images are shown in **Supplementary Figure 3**.

To expand our multiplex IHC platform, we established an HNSCC-targeting multiplex IHC panel assembling candidate biomarkers derived from our systematic review and meta-analysis. Based on a chromogenic sequential IHC method enabling analysis of more than 12 proteins in one tissue section (7), a 14-biomarker multiplex IHC panel was assembled from β-catenin, DKK1, PINCH1, ADAM10, and TIMP1 used as prognostic markers. In addition to hematoxylin staining, HIF1α as a hypoxia marker, ZFX as a transcriptional regulator of self-renewal and stemness, ZEB2 as an epithelial to mesenchymal transformation (EMT) marker, and Ki67 as a proliferation marker were included in the panel, considering the functional significance of these markers. To simultaneously analyze tumor microenvironment-related factors, the panel also included pan-cytokeratin (pCK) as an epithelial marker, CD3 and CD68 as immune markers, and alpha smooth muscle actin (aSMA) as a fibroblastic and vascular endothelial marker (**Figure 5A**). Negative controls were used for confirmation of complete antibody and signal stripping by omitting the primary antibody in the next cycle (data not shown). This panel enabled simultaneous evaluation of tumor, immune, and stroma biomarkers in a single slide, visualizing the opposite distribution of HIF1α and TIMP1 in the intratumor and invasive margins, respectively, (**Figures 5A, B** **)** and accumulation of immune markers (CD3 and CD68) and stroma markers (aSMA) in adjacent non-malignant regions (**Figure 5C** and **Supplementary Figure 5** **).**

**Figure 6:**
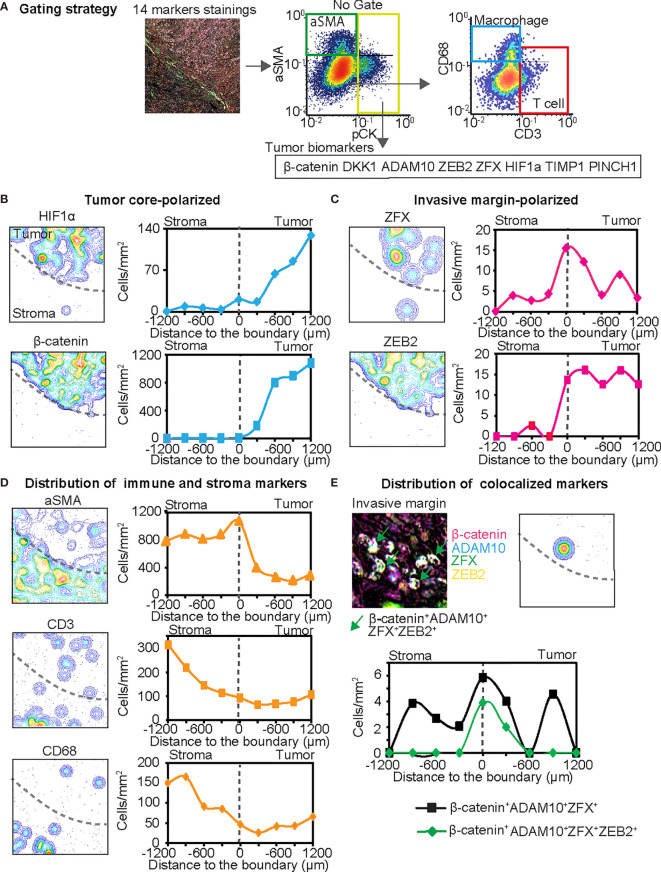
The 14-biomarker multiplex IHC panel quantitatively assesses intratumoral heterogeneity of HNSCC. **(A)** The gating strategy used for single cell-based chromogenic signal intensity is shown where cell size/area, and location are utilized for density plots similar to flow cytometry. X and y axes were shown on a logarithmic scale. The gating strategies identified cell populations of pCK^+^ tumor cells with selected tumor cell biomarkers such as β-catenin, DKK1, ADAM10, ZEB2, ZFX, HIF1α, TIMP1, and PINCH1, together with aSMA^+^ cells (pCK^−^aSMA^+^), T cells (pCK^−^aSMA^−^CD3^+^) and macrophages (pCK^−^aSMA^−^CD68^+^) in a single tissue. **(B–D)** Left panels depict contour mapping based on cell density of each marker. Dash lines present the invasive margin between tumor cell nests and stroma. Right panels depict cell densities of identified lineages in every 200 µm-width from stroma to the tumor side. Tumor cell biomarkers are classified into tumor core-polarized **(B)**, and invasive margin-polarized markers, in comparison with immune and stroma markers **(D)**. **(E)** IHC image and pixel density contour graph indicate distribution of colocalized markers at the invasive margin. Line graph show two colocalized markers. Black line shows β-catenin^+^ DKK1^+^ ADAM10^+^ ZFX^+^ cells and green line showed β-catenin^+^ DKK1^+^ ADAM10^+^ ZFX^+^ ZEB2^+^ cells.

Next, a single quantitative assessment was performed using an image cytometry (**Figure 6A**). Since image cytometry analysis enables single-cell analysis with preserved spatial information (62), cell densities and phenotypes of cancer, immune and stromal cells were spatially evaluated at different proximities to the invasive margin of stroma *versus* intratumor regions (**Figures 6B–E**). Spatial analysis revealed that some tumor cell biomarkers exhibited polarized localization in the tissue structure. For example, increased expression of HIF1α was observed in a distant area from the invasive margin (**Figure 6B**), suggesting the presence of hypoxic conditions at the center of the tumor. In contrast, strong expression of ZFX was observed at the invasive margin (**Figure 6C** **)**, suggesting the presence of heterogeneity in increased survival and self-renewing capabilities of tumor cells *via* ZFX (63). The expression of β-catenin and ZEB2 exhibited characteristic polarized localization in the center and invasive margin, respectively (**Figures 6B, C** **)**. Simultaneously, spatial analysis of T cells (CD3^+^), tumor-associated macrophages (CD68^+^), and aSMA^+^ cells revealed heterogeneity of immune and stromal cell densities (**Figure 6D**).

Furthermore, this panel revealed the potential colocalization of multiple tumor cell markers such as β-catenin, ADAM10, ZFX, and ZEB2 (**Figure 6E**). Interestingly, ZEB2, an EMT marker, was strongly colocalized with Wnt/β-catenin related markers at the invasive margin (**Figure 6E**). Given that the Wnt/β-catenin pathway is associated with cancer cell aggressiveness, and ZEB2 is presumably related to metastatic capability, multiple biomarker-positive cancer cells might be related to the presence of aggressive subpopulations of cancer cells, which are potentially localized at the invasive margin of HNSCC tumors. Taken together, these findings indicate that our 14-biomarker multiplex IHC panel provides a platform for the quantitative assessment of intratumor heterogeneity based on multiple tissue biomarkers identified by a systematic review and meta-analysis.

## Discussion

In this study, we developed a 14-marker multiplexed imaging panel for prognostic biomarkers identified by a systematic review and meta-analysis of prognostically and functionally-relevant IHC-based biomarkers in laryngeal and pharyngeal SCC. IHC analysis using this 14-marker multiplexed panel revealed intratumor heterogeneity that was especially related to an aggressive subpopulation associated with Wnt/β-catenin pathway in HNSCC.

While some of the identified biomarkers such as p53 and Ki-67 are shared among many other cancer types (64–68), our systematic review of laryngeal and pharyngeal HNSCC characteristically identified the prognostic significance of Wnt/β-catenin pathway-related markers such as β-catenin, DKK1, ADAM10, and ZFX (**Table 1** and **Figure 4**) (27, 47, 58, 67, 68). This trend was more pronounced in laryngeal SCC as observed in **Supplementary Figure 4**. These Wnt/β-catenin-related markers are involved in a wide range of functional categories in the Reactome pathway analysis (**Figure 3** and **Supplementary Figure 2**), suggesting that activated β-catenin might be related to disease aggressiveness of HNSCC, particularly in laryngeal SCC.

Although the importance of intratumor heterogeneity has been increasingly acknowledged, the vast majority of IHC-based studies have not considered the significance of heterogeneous expression of tumor biomarkers. Intratumor heterogeneity can be associated with poor clinical outcomes due to the presence of subclones of cancer cells with aggressive characteristics, such as rapid proliferation, metastatic potential, and therapeutic resistance (2). Recent studies based on single-cell RNA sequencing have revealed that HNSCC has remarkable intratumor heterogeneity in terms of clonal diversity (69) and locally activated partial EMT program at the leading edge of primary tumors (70). In agreement with these findings, our multiplex IHC analysis demonstrated the localization of aggressive cancer cell populations expressing Wnt/β-catenin related markers and ZEB2 in the invasive margin (**Figure 6E**). Given that TIMP1 and macrophages were reported to induce the expression of EMT transcriptional factors, including ZEB2 (26, 71), increased expression of TIMP1 and CD68 in the invasive margin might be related to the regulation of EMT and metastatic potential. Multiplex IHC-based simultaneous detection in a single tissue enables quantitative evaluation of multiple overlapping biomarkers together with spatial distribution in the tumor architecture, contributing to the understanding of intratumor heterogeneity of HNSCC.

Immune checkpoint blockade has gained increasing importance in the treatment of HNSCC, and many studies have investigated the association between specific immune cell populations and clinical outcomes of HNSCC (72, 73). Despite the single-case analysis in our study, increased expression of β-catenin in the center of the tumor might be associated with the low density of CD3^+^ T cells (**Figure 6D**), which is potentially associated with T-cell exclusion mechanisms driven by Wnt/β-catenin pathway activation (74). These findings suggest that our 14-biomarker multiplex IHC panel can serve as a platform to characterize the intratumor heterogeneity of cancer cell phenotypes in association with immune cell densities.

In this study, we observed discrepancies between IHC-based results and transcriptomic analyses in terms of prognostic significance. Notably, 15 out of 45 biomarkers exhibited poor prognostic significance in IHC studies, but not in transcriptomic analysis in TCGA (**Table 1** and **Figure 4**), leading to the notion that transcriptomic profiles did not necessarily predict protein expression (75). These observations provide a rationale for revisiting IHC-based proteomics, which can provide a clinically applicable approach for analyzing the intensity and functional properties of protein expression without significant cost (76).

Although extensive research was performed, a potential limitation of this study is publication bias. Due to a great diversity of IHC biomarkers, identified biomarkers in our study were based on a small number of the original studies (**Figure 2**), in analogous to previous IHC meta-analyses (64, 66). To avoid bias due to any potential heterogeneity, we utilized TCGA database to validate the prognostic significance of the identified biomarkers (**Figure 4**). Another limitation in this study is the lack of subgroup analyses stratified by the HNSCC subsites such as larynx, oropharynx, and hypopharynx. Since we observed some interesting differences between laryngeal and pharyngeal SCC (**Supplementary Figure 4**), further studies are required for exploring subsite-specific biomarkers associated with clinical outcomes.

In summary, the present study, based on a systematic review and meta-analysis of laryngeal and pharyngeal SCC, identified IHC-based prognostic biomarkers. Established multiplex IHC panels enable assessment of intratumor heterogeneity of HNSCC, which is particularly related to activation of the Wnt/β-catenin pathway. In combination with other single-cell and bulk tumor analyses, our tissue-based biomarker analysis can provide new insights into diagnostic and treatment strategies for HNSCC.

## Data Availability Statement

The original contributions presented in the study are included in the article/**Supplementary Material**. Further inquiries can be directed to the corresponding author.

## Ethics Statement

The studies involving human participants were reviewed and approved by The Institutional Review Board at Kyoto Prefectural University of Medicine (ERB-C-43-4). The patients/participants provided their written informed consent to participate in this study.

## Author Contributions

JM performed manuscript preparation. JM and TT performed data analysis. JM, MS, and KK collected data and performed IHC. TT conceived of the research and designed research studies. TT, MT, GO, and AA obtained research funding. TT, GO, and AA participated in sample preparation. HO and KI provided digital analytic support for multiplex IHC. TT and SH supervised this study. All authors contributed to the article and approved the submitted version.

## Funding

This work was supported by grants from the Japanese ministry of education, culture, sports, science and technology (17H07016, 19K18814, 19K18739, and 19K23893), and the public promoting association Asano foundation for studies on medicine, and by the research promotion award from the Oto-Rhino-Laryngological Society of Japan, Inc. The funder was not involved in the study design, collection, analysis, interpretation of data, the writing of this article or the decision to submit it for publication.

## Conflict of Interest

HO is an employee for SCREEN Holdings Co., Ltd. KI has received research funding from SCREEN Holdings Co., Ltd.

The remaining authors declare that the research was conducted in the absence of any commercial or financial relationships that could be construed as a potential conflict of interest.

## Publisher’s Note

All claims expressed in this article are solely those of the authors and do not necessarily represent those of their affiliated organizations, or those of the publisher, the editors and the reviewers. Any product that may be evaluated in this article, or claim that may be made by its manufacturer, is not guaranteed or endorsed by the publisher.
