# Supplementary material for: A 14-Marker Multiplexed Imaging Panel for Prognostic Biomarkers and Tumor Heterogeneity in Head and Neck Squamous Cell Carcinoma
Source: Front Oncol. 2021 Aug 19;11:713561. doi: 10.3389/fonc.2021.713561 (PMC8417535; doi:10.3389/fonc.2021.713561)
Supplement: Supplementary file 1 [file DataSheet_1.docx]

**Supplementary information**

Details of representative markers classified by hallmarks

Angiogenesis

EGF-like D7
EGF-like D7 (Egfl7) is initially identified as a novel epidermal growth factor (EGF) domain gene and specifically expressed by endothelial cells. Egfl7 is found to involve in the process of gradual separation and proper spatial arrangement of the angioblasts and plays a crucial role in vascular tubulogenesis. As a novel proangiogenic factor, Egfl7 is essential for angiogenesis during embryonic development and is upregulated during tumorigenesis. Recently, Egfl7 in LSCC is reported to up-regulate tumor grade and lymph node metastasis^1^.

HIF1α

Hypoxia-inducible factor 1 (HIF1) is a transcription factor, which plays a central role in biologic processes under hypoxic conditions, especially concerning tumor angiogenesis. HIF1α is the relevant, oxygen-dependent subunit and its overexpression is associated with a poor prognosis in a variety of malignant tumors^2^. High HIF1α expression is significantly associated with worse overall survival of glottic squamous cell carcinoma patients^3^.

Evading growth suppressor

p63

P63 is a recently cloned p53-related gene mapping to chromosome 3q27–29, which shares structural and functional homologies with the p53 family of transcription factors. It is capable of binding DNA, transactivating p53- responsive genes, and inducing apoptosis^4^. High p63 is significantly associated with patients’ poor overall survival and disease-free survival^4^.

Genome instability & mutation

MAGE-A9

The MAGE-A9 gene, a multicopy member of the MAGEA gene family is an independently acquired X-linked gene that encodes a Cancer/Testis antigen. MAGEA-9 is known to involve the regulation of gene expression, DNA methylation, and cell apoptosis^5^. Additionally, MAGE-A9 plays a role in regulating stemness characteristics in EpCAM^+^ cells. EpCAM is a WNT/β-catenin signaling target gene, thus MAGE-A9 may function in the context of the activation of β-catenin signaling^6^. MiRNAs are important post‐transcriptional regulators of gene expression and are involved in multiple biological processes, including tumorigenesis and metastasis. MiR‐143‐3p is reported to be involved in regulating the MAGE‐A9 expression^7^. High expression of MAGE-A9 in LSCC tissues is associated with a poor prognosis in LSCC patients^7^.

Immune evasion

β-catenin

β-catenin is a multifunctional protein with a central role in physiological homeostasis. The Wnt/β-catenin signaling pathway certifies to regulate cell motility and confer cellular metastasis in cancer^8^. Besides, β-catenin promotes the progression of tumors via suppressing the T-cell responses^9^. The signaling activity of β-catenin is mediated through its interaction with the T cell factor (TCF) family of transcription factors and subsequent activation of target genes. Overexpression of β-catenin significantly reduces OS time of laryngeal squamous cell carcinoma patients^10^.

Invasion & metastasis

DKK1

Dickkopf-1 (DKK1), a secreted protein and Wnt signaling pathway inhibitor is highly expressed in many carcinomas. High expression of DKK1 is related to lymphatic metastasis and indicates poor prognosis in laryngeal squamous cell carcinoma patients after surgery^11^.

LAMP3

Lysosomal associated membrane protein 3 (LAMP3) is a newly identified hypoxia regulated and TP53 downstream target gene involved in hypoxia-induced therapy resistance and metastasis. High LAMP3 is significantly associated with patients’ poor overall survival^4^.

ZEB2

ZEB2 is associated with EMT proposed to be involved in this key step of the progression of different types of tumor. ZEB2 expression is significantly increased in tumor tissue compared with non-carcinoma tissue and is directly associated with the status of lymph node metastases, T stage and tumor cell differentiation in LSCC. Positive ZEB2 expression is associated with a poor prognosis in patients with LSCC^10^.

Metadherin
Metadherin (MTDH) is a single-pass transmembrane protein, and its gene is located on chromosome 8q22. Its diverse functions include modulating cell proliferation, angiogenesis, drug resistance, and stem cell transformation. Recent study suggests that MTDH could promote EMT, which would lead to enhanced invasion and metastasis of HNSCC cells^12^.

Metabolism

GpX1

GPXs, a family of selenoprotein-containing antioxidant enzymes, can efficiently eliminate hydrogen peroxide and other ROS in the body and protect cells from the oxidative DNA damage. Nevertheless, the GPXs expressions appear obviously aberrant in the pathological process of cancers. High Gpx1 expression level is an independent poor prognostic factor of survival in LSCC patients^13^.

Replicable immortality

MCM7

Minichromosome maintenance proteins (MCMs) belong to a family of 6 highly conserved and highly homologous proteins (MCM2–7). MCM proteins 2 to 7 form a functional hexameric complex, constituting an important part of the pre-recognition complex of proteins present at DNA replication origins during the G1 phase of the cell cycle. MCM proteins represent a reliable marker of cell cycle entry as their expression demonstrate in cells remaining in the cell cycle, whereas loss of MCM expression reflects the resting state of the cells. MCM7 labeling index values are directly associated with the risk of progression and death in patients with LSCC^14^.

NDRG3

The NDRG protein family consists of four members, NDRG1–4, which are speculated to play complicated roles in diverse biological processes. NDRG3 promoted angiogenesis, cell proliferation, and anti-apoptosis by activating the RAF–ERK pathway. Moreover, NDRG3 expression is elevated in LSCC tissues and might be associated with LSCC progression^15,16^.

Resisting cell death

PINCH1

Particularly interesting new cysteine-histidine-rich protein (PINCH) provides a link between the ECM and the actin cytoskeleton at integrin adhesion sites. PINCH binds to integrin-linked kinase (ILK) forming the heterotrimeric ILK-parvin-PINCH (IPP) complex that functions as a protein scaffold and signaling hub with a key role in focal adhesion assembly and integrin signaling. PINCH also regulates cell survival in part through effects on ILK and Akt signaling. Increased expression of PINCH1is reported in human cancer promoting cancer cell migration, invasion, and apoptosis resistance^17^.

SOX2

SOX2 is an essential transcription factor, which not only has role during neurogenesis and embryonic foregut development, but also allows reprogramming of adult cells to pluripotent stem cells. SOX2 promotes proliferation, clonogenicity, and tumorigenicity of cervical cancer cells, and similar results are reported in breast cancer. Moreover, SOX2 overexpression is associated with progression and poor prognosis in LSCC^18^.

ZFX

Zinc finger protein X-linked (ZFX) belongs to the zinc finger protein super family. ZFX plays an important role in various biological processes, such as gene expression, cell differentiation and embryonic development. As a regulation factor, ZFX participates in cell cycle control, self-renewal in several sorts of stem cell, cell proliferation and the regulation of cancer related gene or pathway. Moreover, ZFX expression promotes β-catenin nuclear translocation and enhanced its transcriptional activity. Patients with high expression of ZFX are shown to have a lower overall survival rate than patients with low expression of ZFX^19^.

Sustaining proliferation

ASCL1

Achaete-scute complex homo-logue-1 (ASCL1) plays an important role in a series of cellular processes such as intracellular transportation and cell division. Silencing ASCL1 expression suppress the proliferation of both cancer and neuroendocrine tumor. Moreover, ASCL1 can facilitate EMT, cell proliferation, migration, and invasion as well as cell-cycle arrest and induce apoptosis of laryngeal carcinoma by binding to Wnt inhibitor DKK1^20^.

SHIP2

SH2-containing 5′-inositol phosphatase-2 (SHIP2) regulates multiple oncogenic signaling pathways by dephosphorylating PIP3 to produce phosphatidylinositol-3, 4-bisphosphate (PI-3,4-P2). These pathways include the phosphatidylinositol 3-kinase (PI3K) and Akt cascade as well as the Ras and mitogen-activated protein (MAP) kinase cascade which, in turn, regulate cell proliferation, differentiation, and apoptosis. SHIP2 protein is upregulated in human LSCC and SHIP2 overexpression is significantly correlated with tumor malignant progression and poor survival in patients with LSCC^21^.

γ-H2AX

γ-H2AX (histone H2AX) plays a key role in the repair of DNA damage associated with double-strand breaks. It synergistically blocks the cell cycle, induces apoptosis, and promotes DNA repair by avoiding the accumulation of damaged DNA, maintaining genetic stability, and preventing cell transformation. High γ-H2AX expression is negatively correlated with overall survival in laryngeal squamous cell carcinoma patients^22^.

Tumor promoting inflammation

ADAM10

The ADAMs are a family of proteins that contain an N-terminal prodomain preceding a metalloproteinase domain, a disintegrin or integrin-binding domain, a cysteine-rich region, a transmembrane domain and an intracellular domain. ADAM10 modulates the nuclear translocation of β-catenin through shedding of cadherin E, resulting in enhanced expression of cyclin D1 and c-Myc, thus leading to the promotion of proliferation. High ADAM10 expression is involved in the aggressive malignant phenotype and poor prognosis of laryngeal carcinoma^23^.

COX-2

COX-2 can rapidly induce a response to tumor-promoting cytokines and growth factors through pathological pathways that affect mitosis, cell adhesion and immune monitoring. COX-2 implicates in promoting tumorigenesis and cancer progression. Oropharyngeal squamous cell carcinoma patients with COX-2 high expression have a worse prognosis than those with low COX-2 expression^24^.

NF-kB

The nuclear factor-κB (NF-κB) is a transcription factor that is retained in the cytoplasm by the inhibitory protein IκB. Phosphorylated IκBα is ubiquitinated and subsequently degraded by the 26S proteasome, resulting in the liberation of NF-κB^25^. On the hand, β-catenin inhibits NF-κB activity, downregulating Fas expression, which may allow cancer cells to escape immune surveillance^26^. High NF-κB is associated with hematologic and lymphatic metastasis of HNSCC^25^.

**References for Supplementary Information**

1. Li J, Chen W, Wang K, Zhu Z, Zhang J, He F, Tang S, Tang Q, Yang X, Li S, Wang Y. Epidermal growth factor-like domain 7 promotes cell proliferation, invasion and serves as a prognostic indicator in human laryngeal squamous cell carcinoma. *Int J Clin Exp Pathol* 2016;9:8899–910.

2. Fillies T, Werkmeister R, van Diest PJ, Brandt B, Joos U, Buerger H. HIF 1-alpha overexpression indicates a good prognosis in early stage squamous cell carcinomas of the oral floor. *BMC Cancer* 2005;5:1–9.

3. Schrijvers ML, Pattje WJ, Slagter-Menkema L, Mastik MF, Gibcus JH, Langendijk JA, Van Der Wal JE, Van Der Laan BFAM, Schuuring E. FADD expression as a prognosticator in early-stage glottic squamous cell carcinoma of the larynx treated primarily with radiotherapy. *Int J Radiat Oncol Biol Phys* 2012;83:1220–6.

4. Qiu X, You Y, Huang J, Wang X, Zhu H, Wang Z. LAMP3 and TP53 overexpression predicts poor outcome in laryngeal squamous cell carcinoma. *Int J Clin Exp Pathol* 2015;8:5519–27.

5. Shen Y, Xu J, Yang X, Liu Y, Ma Y, Yang D, Dong Q, Yang Y. Evidence for the involvement of the proximal copy of the MAGEA9 gene in Xq28-linked CNV67 specific to spermatogenic failure. *Biol Reprod* 2017;96:610–6.

6. Wei Y, Wang Y, Gong J, Rao L, Wu Z, Nie T, Shi D, Zhang L. High expression of MAGE-A9 contributes to stemness and malignancy of human hepatocellular carcinoma. *Int J Oncol* 2018;52:219–30.

7. Han L, Jiang B, Wu H, Zhang S, Lu X. Expression and prognostic value of MAGE-A9 in laryngeal squamous cell carcinoma. *Int J Clin Exp Pathol* 2014;7:6734–42.

8. Zha L, Zhang J, Tang W, Zhang N, He M, Guo Y, Wang Z. HMGA2 elicits EMT by activating the Wnt/β-catenin pathway in gastric cancer. *Dig Dis Sci* 2013;58:724–33.

9. Shang S, Hua F, Hu ZW. The regulation of β-catenin activity and function in cancer: Therapeutic opportunities. *Oncotarget* 2017;8:33972–89.

10. Zhu GJ, Song PP, Zhou H, Shen XH, Wang JG, Ma XF, Gu YJ, Liu DD, Feng AN, Qian XY, Gao X. Role of epithelial-mesenchymal transition markers E-cadherin, N-cadherin, β-catenin and ZEB2 in laryngeal squamous cell carcinoma. *Oncol Lett* 2018;15:3472–81.

11. Shi Y, Gong H-L, Zhou L, Tian J, Wang Y. Dickkopf-1 is a novel prognostic biomarker for laryngeal squamous cell carcinoma. *Acta Otolaryngol* 2014;134:753–9.

12. Yu C, Liu Y, Tan H, Li G, Su Z, Ren S, Zhu G, Tian Y, Qiu Y, Zhang X. Metadherin regulates metastasis of squamous cell carcinoma of the head and neck via AKT signalling pathway-mediated epithelial–mesenchymal transition. *Cancer Lett* 2014;343:258–67.

13. Zhang Q, Xu H, You Y, Zhang J, Chen R. High Gpx1 expression predicts poor survival in laryngeal squamous cell carcinoma. *Auris Nasus Larynx* 2018;45:13–9.

14. Almadori, Giovanni; Lauriola, Libero; Coli, Antonella; Bussu, Francesco; Gallus, Roberto; Scannone, Domenico; Valentini, Vincenzo; Paludetti, Gaetano; Carey, Thomas E.; Ranelletti FO. Minichromosome maintenance protein 7 and geminin expression: Prognostic value in laryngeal squamous cell carcinoma in patients treated with radiotherapy and cetuximab. *Head Neck* 2017;39:684–93.

15. Ma J, Liu S, Zhang W, Zhang F, Wang S, Wu L, Yan R, Wu L, Wang C, Zha Z, Sun J. High expression of NDRG3 associates with positive lymph node metastasis and unfavourable overall survival in laryngeal squamous cell carcinoma. *Pathology* 2016;48:691–6.

16. Lee GY, Shin SH, Shin HW, Chun YS, Park JW. NDRG3 lowers the metastatic potential in prostate cancer as a feedback controller of hypoxia-inducible factors. *Exp Mol Med* 2018;50.

17. Tsinias G, Nikou S, Papadas T, Pitsos P, Papadaki H, Bravou V. High PINCH1 expression in human laryngeal carcinoma associates with poor prognosis. *Anal Cell Pathol* 2018;2018.

18. Tang X, Shen X, Li L, Zhang Y, Chen G. SOX2 overexpression correlates with poor prognosis in laryngeal squamous cell carcinoma. *Auris Nasus Larynx* 2013;40:481–6.

19. Yang F, Ma H, Feng L, Lian M, Wang R, Fan E, Fang J. Zinc finger protein x-linked (ZFX) contributes to patient prognosis, cell proliferation and apoptosis in human laryngeal squamous cell carcinoma. *Int J Clin Exp Pathol* 2015;8:13886–99.

20. Ma H, Du X, Zhang S, Wang Q, Yin Y, Qiu X, Da P, Yue H, Wu H, Xu F. Achaete-scute complex homologue-1 promotes development of laryngocarcinoma via facilitating the epithelial-mesenchymal transformation. *Tumor Biol* 2017;39.

21. Zhou X, Liu Y, Tan G. Prognostic Value of Elevated SHIP2 Expression in Laryngeal Squamous Cell Carcinoma. *Arch Med Res* 2011;42:589–95.

22. Zhang J, Cheng L, Zhou L. Prognostic significance of γ-H2AX in laryngeal squamous cell carcinoma after surgery. *Chin Med J (Engl)* 2014;127:2664–7.

23. You B, Gu M, Cao X, Li X, Shi S, Shan Y, You Y. Clinical significance of ADAM10 expression in laryngeal carcinoma. *Oncol Lett* 2017;13:1353–9.

24. Chen YF, Luo RZ, Li Y, Cui BK, Song M, Yang AK, Chen WK. High expression levels of COX-2 and P300 are associated with unfavorable survival in laryngeal squamous cell carcinoma. *Eur Arch Oto-Rhino-Laryngology* 2013;270:1009–17.

25. Yan M, Xu Q, Zhang P, Zhou X jian, Zhang Z yuan, Chen W tao. Correlation of NF-κB signal pathway with tumor metastasis of human head and neck squamous cell carcinoma. *BMC Cancer* 2010;10.

26. Deng J, Miller SA, Wang HY, Xia W, Wen Y, Zhou BP, Li Y, Lin SY, Hung MC. β-catenin interacts with and inhibits NF-κB in human colon and breast cancer. *Cancer Cell* 2002;2:323–34.
