# Supplementary material for: A 14-Marker Multiplexed Imaging Panel for Prognostic Biomarkers and Tumor Heterogeneity in Head and Neck Squamous Cell Carcinoma
Source: Front Oncol. 2021 Aug 19;11:713561. doi: 10.3389/fonc.2021.713561 (PMC8417535; doi:10.3389/fonc.2021.713561)

Legends for Supplementary Figures

Figure S1. Forest plots of the effects of five biomarkers evaluated by two or more independent studies on laryngeal and pharyngeal HNSCC survival on a random effect model. (**A–E**) Forest plots of hazard ratio (HR) for overall survival (OS) of metadherin (A), ZEB2 (B), p53 (C), Ki-67 (D), and p16 (E) are shown based on a random effect model. Bars present 95% confidence intervals (CI) of HR, and the center of the lozenge gives the combined HR. Combined fixed effect HRs and tests for heterogeneity (I^2) are based on Der Simonian-Laird method.

Fig. S2. An overview for functional significance is visualized using the Reactome pathway database. A detailed Voronoi diagram is presented in support of Figure 3. HBEGF, TP63, HIF1α, MAGEA9, CTNNB1, DKK1, Kif2a, LAMP1, LAMP3, TIMP1, TACSTD2, ZEB2, MTDH, GpX1, RPS6, MCM7, NDRG3, ABCG2, PINCH1, SOX2, ZFX, ASCL1, PHF8, PTOV1, SHIP2, ZNF703, H2AX, TNFSF13, ADAM10, PTGS2, and NFKB1genes corresponding to the 31 IHC biomarkers identified by the systematic review and metaanalysis are utilized as a reference dataset, and the results are visualized as a Voronoi diagram based on ReacFoam function (https://reactome.org). Significantly enriched pathways associated with dataset of genes are shown with a color scale from dark to light yellow.

Fig. S3. Prognostic significance of the selected IHC biomarkers in TCGA. (A–X) Kaplan-Meier analyses of overall survival (OS) in the laryngeal and pharyngeal HNSCC cohort of the Cancer Genome Atlas (TCGA) (N = 205) stratified by gene expression of the identified biomarkers were shown. Median is used for the cutoff values. Statistical significance is determined using log-rank test. The hallmarks of cancer closely related to each marker are shown.

Fig. S4. Overall survival status stratified by laryngeal and pharyngeal SCC in Fig. 4. (A–G) Kaplan-Meier analyses of overall survival in the laryngeal (n = 123) and pharyngeal (n = 82) SCC cohorts of TCGA stratified by gene expression of the identified biomarkers in **Fig. 4** were shown by primary sites. Median is used for the cutoff values. Statistical significance is determined using log-rank test.

Fig. S5. Single marker IHC images in reference to Fig. 5.
Single-channel images of multiplex cycles are shown in **Fig. 5**. Scale bars = 100 μm.

Supplementary figure1

A.metadherin:overall survival

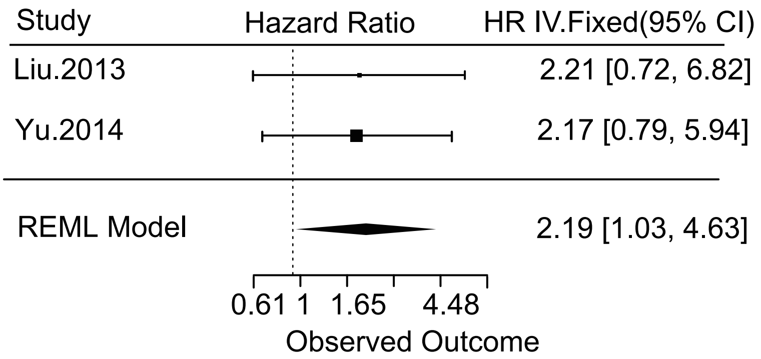

Total(95% CI)
Heterogeneity:Q(df = 1) = 0.0007, p-val = 0.9793 I²=0%
Test for overall effect:Z=2.0443 (P= 0.0409)

D.Ki-67:overall survival

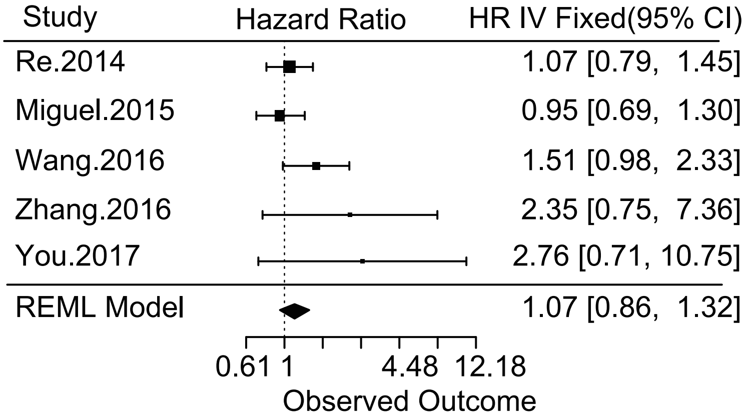

Total(95% CI)
Heterogeneity:Q(df = 4) = 6.2438, p-val = 0.1817 I²=31%
Test for overall effect:Z=1.3522 (P= 0.176)

B.ZEB2:overall survival

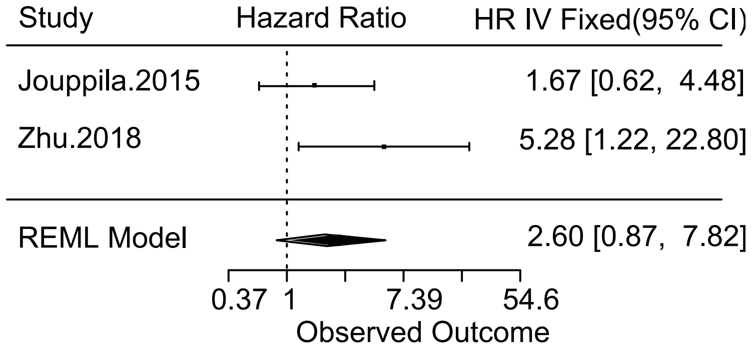

Total(95% CI)
Heterogeneity:Q(df = 1) = 1.6392, p-val = 0.2004 I²=39%
Test for overall effect:Z=1.7033 (P= 0.0885)

E.p16:overall survival

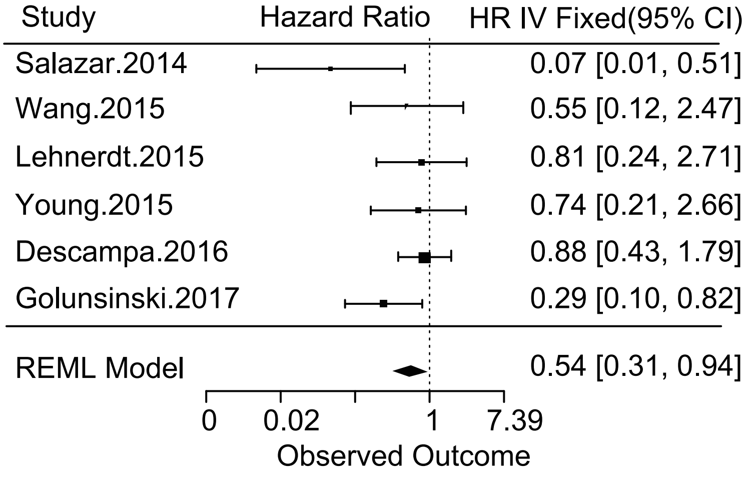

Total(95% CI)
Heterogeneity:Q(df = 5) = 7.737, p-val = 0.1713 I²=27%
Test for overall effect:Z=2.1718 (P= 0.0299)

C.p53:overall survival

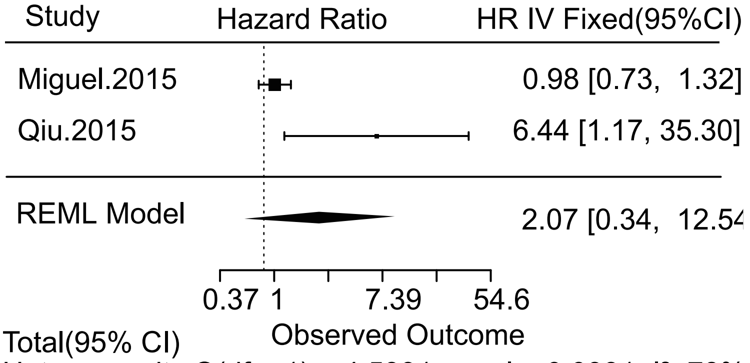

Total(95% CI)
Heterogeneity:Q(df = 1) = 4.5391, p-val = 0.0331 I²=78%
Test for overall effect:Z=0.7929 (P= 0.4278)

Supplementary figure2

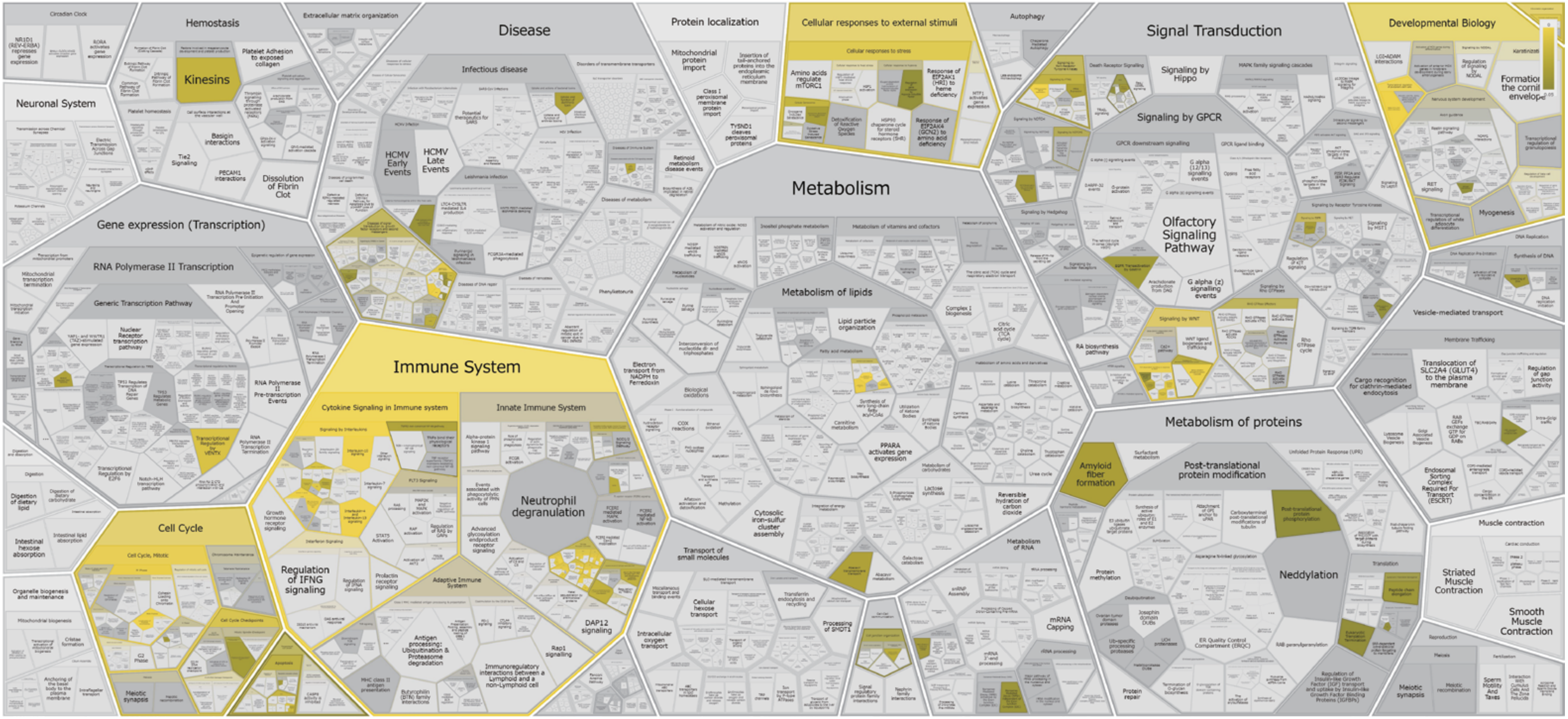

Supplementary figure3-1

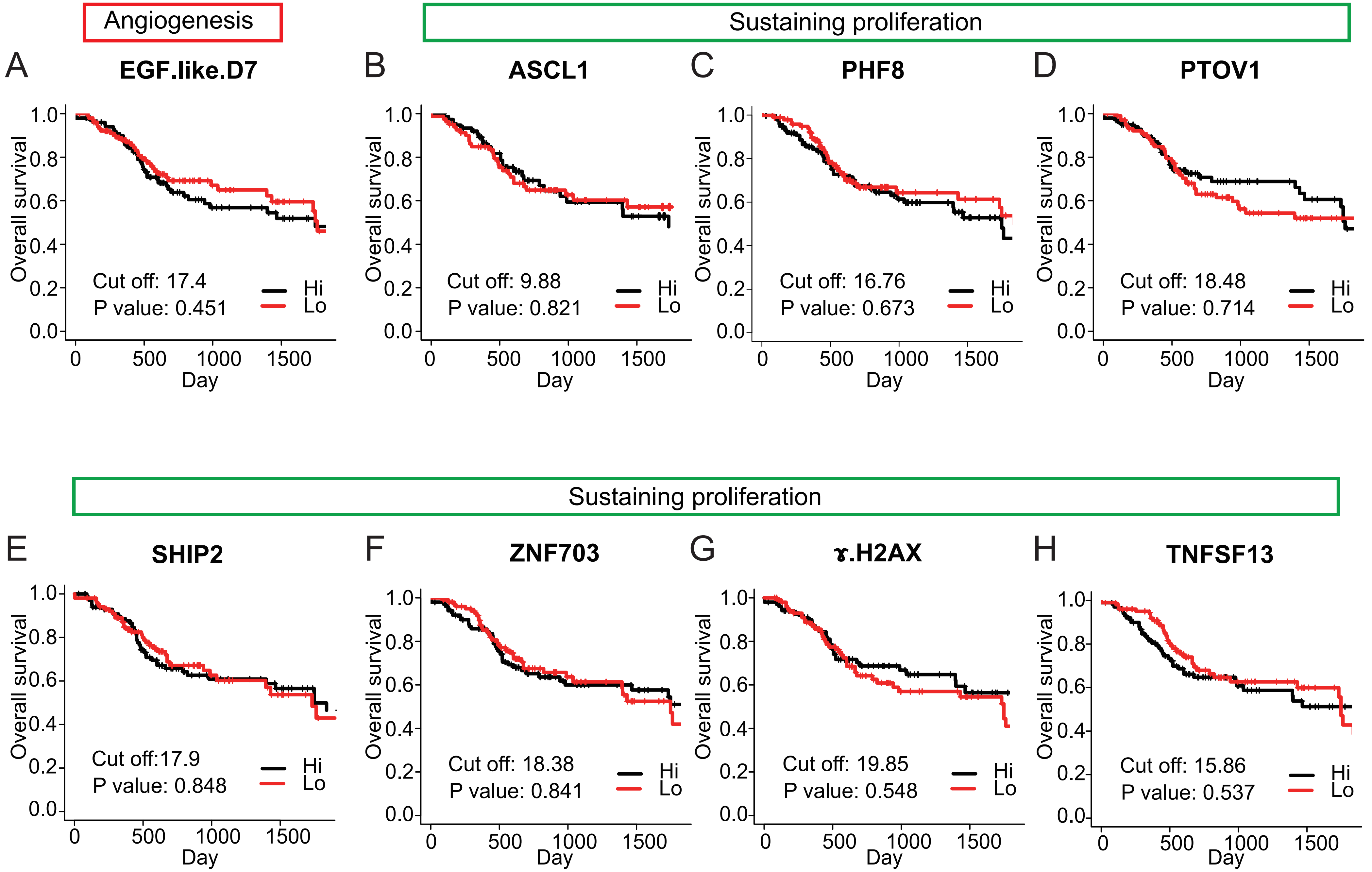

Supplementary figure3-2

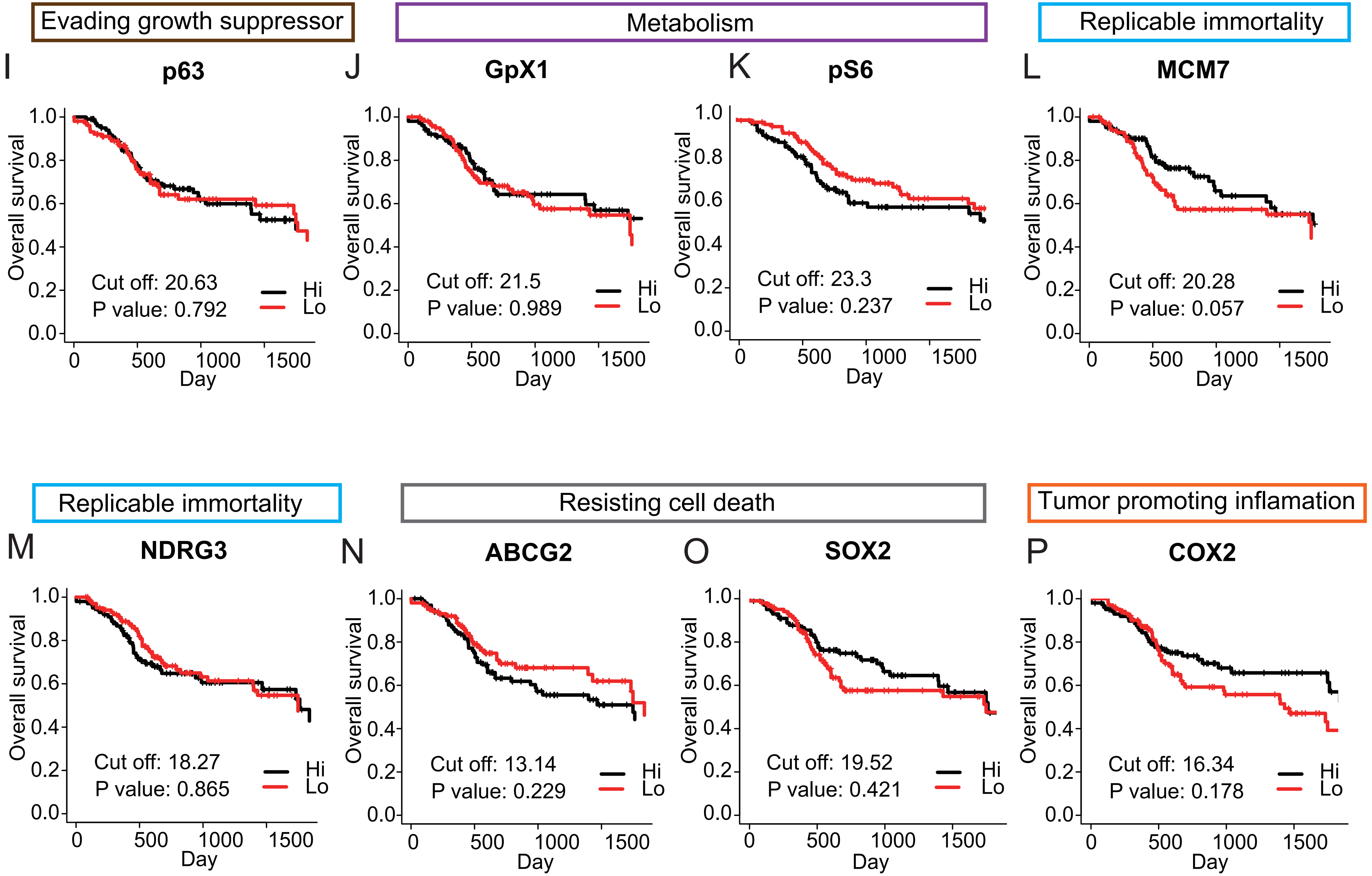

Supplementary figure3-3

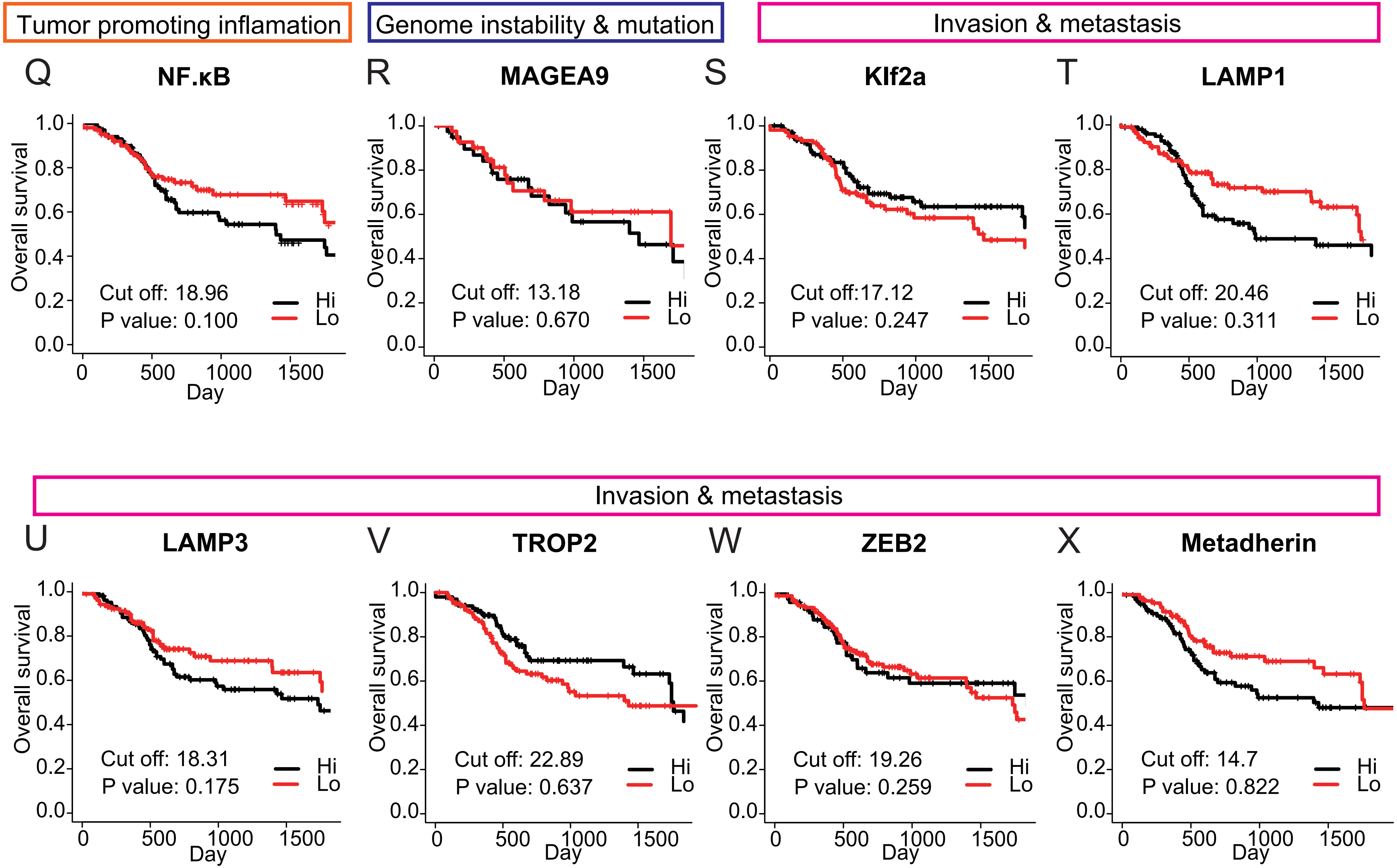

Supplementary figure 4

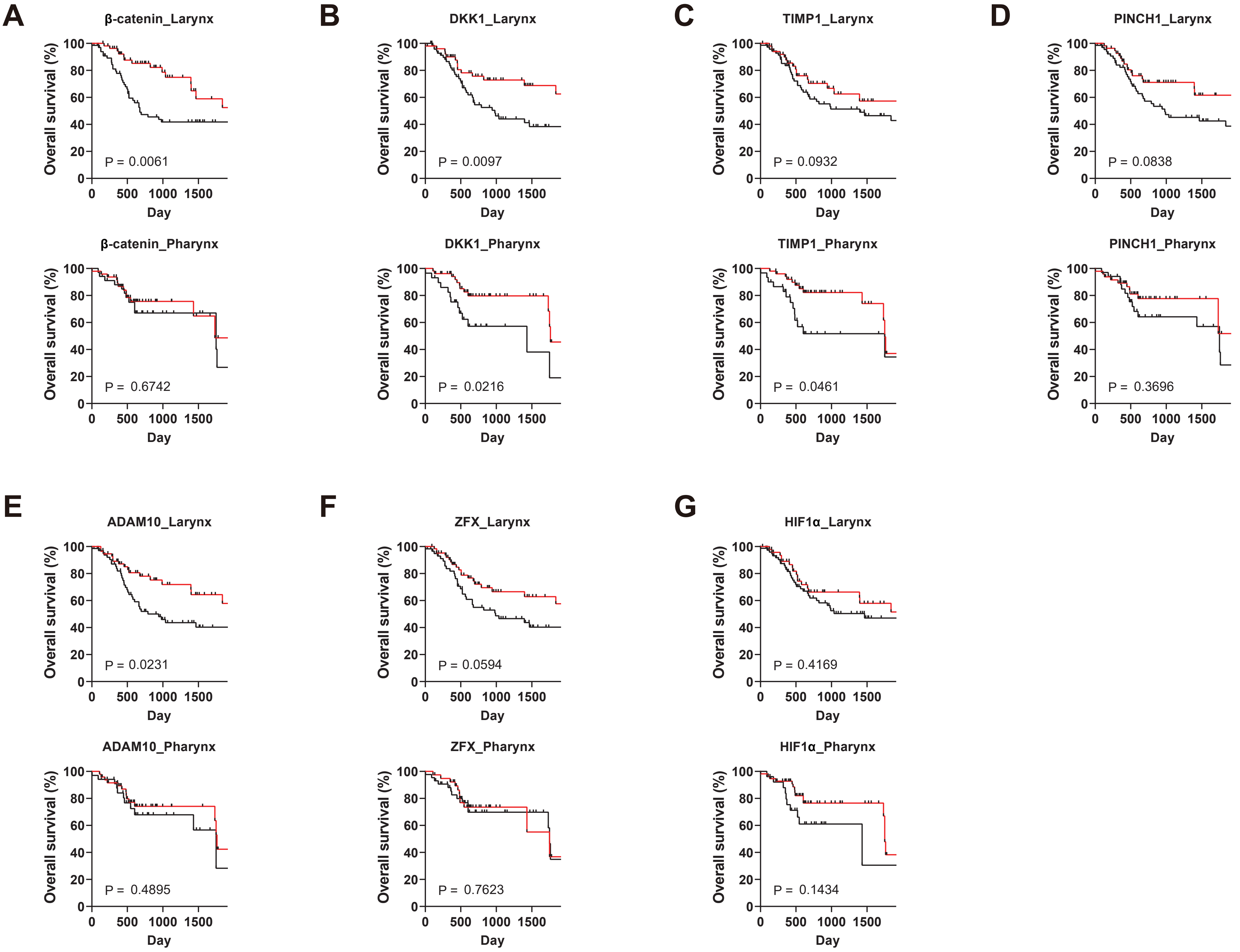

Supplementary figure 5

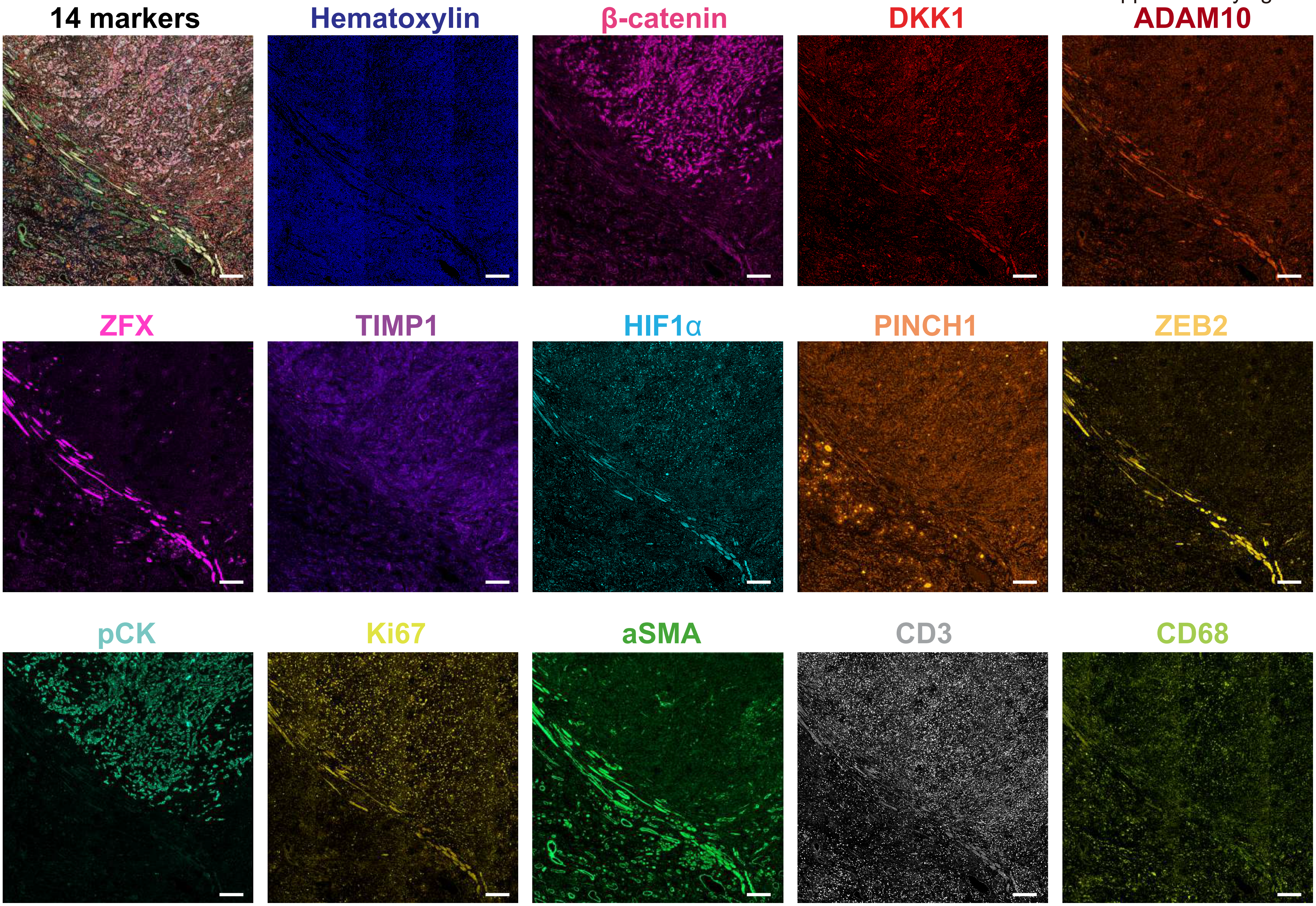

Supplement: Supplementary file 2 [file DataSheet_2.pdf]
