# Supplementary material for: A 14-Marker Multiplexed Imaging Panel for Prognostic Biomarkers and Tumor Heterogeneity in Head and Neck Squamous Cell Carcinoma
Source: Front Oncol. 2021 Aug 19;11:713561. doi: 10.3389/fonc.2021.713561 (PMC8417535; doi:10.3389/fonc.2021.713561)
Supplement: Supplementary file 3 [file Table_1.docx]

**Supplementary Table 1.** **Evaluation criteria modified by REporting recommendations for tumor MARKer prognostic studies (REMARK) criteria**

| 1 | State the marker examined, the study objectives, and any pre-specified hypotheses |
| --- | --- |
| 2 | Describe the characteristics (e.g., disease stage or co-morbidities) of the study patients, including their source and inclusion and exclusion criteria |
| 3 | Describe type of biological material used (including control samples) and methods of preservation and storage |
| 4 | Specify the assay method used and provide (or reference) a detailed protocol, including specific reagents or kits used, quality control procedures, reproducibility assessments, quantitation methods, and scoring and reporting protocols Specify whether and how assays were performed blinded to the study endpoint |
| 5 | State the method of case selection, including whether prospective or retrospective and whether stratification or matching(e.g., by stage of disease or age) was used Specify the time period from which cases were taken, the end of the follow-up period, and the median follow-up time |
| 6 | Specify all statistical methods, including details of any variable selection procedures and other model-building issues, how model assumptions were verified, and how missing data were handled |
| 7 | Clarify how marker values were handled in the analyses; if relevant, describe methods used for cut point determination |
| 8 | Report distributions of basic demographic characteristics (at least age and sex), standard (disease-specific) prognostic variables, and tumor marker, including numbers of missing values |
| 9 | Present univariable analyses showing the relation between the marker and outcome, with the estimated effect (e.g., hazard ratio and survival probability) Preferably provide similar analyses for all other variables being analyzed For the effect of a tumor marker on a time-to-event outcome, a Kaplan-Meier plot is recommended |
| 10 | For key multivariable analyses, report estimated effects (e.g., hazard ratio) with confidence intervals for the marker and, at least for the final model, all other variables in the model |
| 11 | Interpret the results in the context of the pre-specified hypotheses and other relevant studies; include a discussion of limitations of the study |
| 12 | Discuss implications for future research and clinical value |
