# Supplementary material for: A 14-Marker Multiplexed Imaging Panel for Prognostic Biomarkers and Tumor Heterogeneity in Head and Neck Squamous Cell Carcinoma
Source: Front Oncol. 2021 Aug 19;11:713561. doi: 10.3389/fonc.2021.713561 (PMC8417535; doi:10.3389/fonc.2021.713561)
Supplement: Supplementary file 4 [file Table_2.docx]

**Table S2**. **Sequential IHC panel information.**

|  | Cycle1 | Cycle2 | Cycle3 | Cycle4 | Cycle5 | Cycle6 | Cycle7 |
| --- | --- | --- | --- | --- | --- | --- | --- |
| Primary Ab | Hem | ZEB2 | HIF1α | β-catenin | CD3 | DKK-1 | PINCH1 |
| Clone/Product# | S3301 | HPA003456 | ab114977 | ab16051 | SP7 | ab61034 | MABT162 |
| Vender | Dako | Merck | Abcam | Abcam | Thermo scient | Abcam | Merck |
| Concentration |  | 1/75 | 1/75 | 1/300 | 1/100 | 1/100 | 1/40 |
| Reaction time | 1min | RT, 30min | RT, 30min | RT, 30min | RT, 30min | RT, 30min | RT, 30min |
| Secondary Ab |  | Anti-rabbit | Anti-rabbit | Anti-rabbit | Anti-rabbit | Anti-rabbit | Anti-mouse |
| Reaction time |  | RT, 30min | RT, 30min | RT, 30min | RT, 30min | RT, 30min | RT, 30min |
| AEC reaction time |  | 20min | 20min | 20min | 20min | 20min | 20min |
|  |  |  |  |  |  |  |  |
|  |  |  |  |  |  |  |  |
|  | Cycle8 | Cycle9 | Cycle10 | Cycle11 | Cycle12 | Cycle13 | Cycle14 |
| Primary Ab | α-SMA | CD68 | ZFX | ADAM10 | Ki-67 | pCK | TIMP1 |
| Clone/Product# | ab5694 | PG-M1 | HPA003877 | ab19026 | SP6 | AE1/AE3 | ab211926 |
| Vender | Abcam | Abcam | Merck | Merck | Sigma-Aldrich | Abcam | Abcam |
| Concentration | 1/200 | 1/50 | 1/1200 | 1/2000 | 1/2000 | 1/2000 | 1/1000 |
| Reaction time | RT, 30min | RT, 30min | RT, 30min | RT, 30min | RT, 30min | RT, 30min | RT, 30min |
| Secondary Ab | Anti-rabbit | Anti-mouse | Anti-rabbit | Anti-rabbit | Anti-rabbit | Anti-mouse | Anti- rabbit |
| Reaction time | RT, 30min | RT, 30min | RT, 30min | RT, 30min | RT, 30min | RT, 30min | RT, 30min |
| AEC reaction time | 20min | 20min | 20min | 20min | 20min | 20min | 20min |
